# Untreated Hair Dye Effluents Enter the Environment: Are They a Threat to Human Health?

**DOI:** 10.1002/tox.70034

**Published:** 2026-02-02

**Authors:** Letícia Cristina Gonçalves, Matheus Mantuanelli Roberto, Adriana Fabiana Corrêa da Silva, Maria Aparecida Marin‐Morales

**Affiliations:** ^1^ Department of Biology, Institute of Biosciences São Paulo State University (UNESP) Rio Claro São Paulo Brazil; ^2^ University Center of Hermínio Ometto Foundation (FHO) Araras São Paulo Brazil

**Keywords:** cell viability, comet assay, HepG2 cell line, micronucleus test, mitochondrial activity

## Abstract

The effluents generated during the process of hair dyeing exhibit a complex composition, comprising chemical compounds with varying toxicity levels. While the adverse impact of hair dyes on human health is acknowledged, there is a notable absence of studies addressing the toxicity associated with effluents produced during these activities. The primary objective of this study was to assess two effluents emanating from beauty salons after brown hair dyeing: one resulting from hair washing with water, shampoo, and conditioner, referred to as the complete effluent (CE), and the other from washing the dyed hair solely with water, excluding surfactants, referred to as the dye effluent (DE). In vitro bioassays were conducted with the human hepatoma cell line (HepG2/C3A). Cytotoxicity was evaluated through 3‐(4,5‐dimethylthiazol‐2‐yl)‐2,5‐diphenyltetrazolium bromide (MTT) and Trypan Blue tests, while genotoxicity and mutagenicity were assessed by comet assay and cytokinesis‐block micronucleus test, respectively. The cells were exposed for 4 h to various dilutions of the two sampled effluents [100.0% (1); 50.0% (2); 25.0% (3); 12.5% (4); 6.25% (5); 3.125% (6)]. Cytotoxicity was induced by CE‐1, DE‐1, DE‐2, and DE‐3 dilutions as indicated by both assays, whereas CE‐2 dilution exhibited cytotoxicity solely through the MTT assay. These findings suggest impaired cell membrane integrity, permeability, and mitochondrial activity. Nontoxic dilutions (4, 5, and 6) were viable for the comet assay and micronucleus test, revealing genotoxicity without mutagenic potential. Consequently, residual concentrations of effluents were found to induce nonlethal and reparable primary DNA damage. Moreover, the effluents decreased the cytokinesis‐block proliferation index and the cell replication index, indicating interference and arrestment in the cell cycle. These outcomes highlight the potential threat posed by residual concentrations of hair DEs to environmental quality and human health, emphasizing the imperative need for pre‐disposal treatments in salon settings.

## 1 Introduction

Hair dyeing is an ancient technique still widely used to modify or restore hair color. The coloring process involves several chemical additives and complex oxidative reactions inside the hair fiber [1, 2].

Effluents generated in beauty salons vary depending on the hair dyeing process and typically contain dye residues, precursors, couplers, cosmetic additives, and metals like lead, cadmium, chromium, and arsenic [3, 4, 5], as well as hydrogen peroxide and surfactants used in hair washing to remove excess dye [1, 5].

The water demand in beauty salons is substantial, driven by the need for frequent hair washing to ensure complete removal of applied cosmetic products [6]. This complex mixture can alter the characteristics of receiving water bodies, increasing several physicochemical parameters such as organic matter, turbidity, and suspended and dissolved solids [7]. Despite significant effluent production, studies evaluating potential risks to the environment and human health remain scarce [8].

The possible toxic effects of human exposure to hair dyes have been reevaluated by various authors. Adverse effects include systemic reactions by inhalation, oral contact, or percutaneous absorption, such as skin irritations, dermatitis, allergies, sensitization, and carcinogenic action [1, 9].

Beauty salon products pose health risks, especially to professionals continuously exposed to these cosmetics, with limited awareness of the risks due to the diversity of new products [5, 9].

Studies highlight a significant knowledge gap regarding the ecotoxicity of personal care products (PCPs) in aquatic environments. PCPs are more prevalent and at higher concentrations compared to pharmaceuticals but have not received equivalent attention [10]. Existing studies often examine individual compounds without considering the synergistic effects of contaminants in effluents [3, 11].

The lack of ecotoxicological studies on these products also leads to a lack of data on the persistence and biotransformation of hair dye components, making monitoring these by‐products in salon wastewater unfeasible [1, 12]. Thus, there is an urgent need to characterize these compounds in terms of their toxicity and environmental danger, considering their chemical and reactive properties [13].

Beauty salons are growing globally, driven by the ideology of well‐being and happiness, regardless of the country's economic status [14]. Wastewater treatment plants (WWTPs) are generally not designed to eliminate the toxic potential of effluents, leading to environmental issues such as dye accumulation, ecosystem destabilization, and human health damage [3, 15].

Many studies have described the adverse effects of chemical compounds on living organisms using cell line tests, which are sensitive, fast, and reliable in elucidating contaminants' mechanisms of action [16]. Although humans are the main target of concern in toxicity studies, the use of DNA as a biomarker allows the effects to be extrapolated to other living beings. Therefore, it is possible to infer that compounds that react with the DNA of one species have the potential to act similarly in other species [17].

Considering this context, the present study evaluated the cytotoxicity, genotoxicity, and mutagenic potential of two types of effluents from beauty salons using the human hepatocarcinoma cell line (HepG2/C3A). Thus, this research supports a better understanding of the ecogenotoxic potential of beauty salon effluents posthair dyeing.

As sewage and water treatments usually are not effective in removing this class of contaminants, it is important to emphasize the human health risks associated with polluted drinking water. Also, the results presented here can assist regulatory agencies in formulating guidelines for proper management by beauty salons and WWTPs to mitigate impacts on water bodies and, consequently, on One Health [18, 19].

## 2 Materials and Methods

### 2.1 Collection, Storage, and Preparation of Effluent Samples

Considering the worst contamination scenario, this study evaluated raw effluents without undergoing any type of treatment. Two types of effluents resulting from the hair dyeing process were collected at a beauty salon in the city of Rio Claro, State of São Paulo, Brazil: effluent produced during hair washing using only water (dye effluent—DE) and effluent from hair washing involving water, shampoos, and conditioners (complete effluent—CE). The hair coloring involved the use of a brown dye produced by a worldwide company.

After collection, the effluents were individually packaged in plastic containers, labeled, and stored in a freezer at −20°C to prevent potential degradation or alteration of their properties.

For the experimental procedures, the effluent samples were maintained at 37°C, and the pH was adjusted to 7.2 to ensure optimal conditions for cell culture. The original pH values of the samples were 5.14 for CE and 6.16 for DE. From these samples, serial dilutions were prepared (1 = 100.0%; 2 = 50.0%; 3 = 25.0%; 4 = 12.5%; 5 = 6.25%; 6 = 3.125%) using reverse osmosis water, followed by sterilization through a polyethersulfone membrane filter (33 mm diameter and 0.22 μm pore size, Prolab, São Paulo, Brazil).

The cells used in the experiments were exposed to a solution of 20% of the samples (dilutions of DE or CE) and 80% of the culture medium [20]. This ratio was chosen based on data provided by Reifferscheid et al. [21], indicating that the addition of 20% of the sample does not induce cytotoxic and genotoxic effects in exposed cells due to changes in osmolarity.

### 2.2 Culture of HepG2/C3A Cells

The in vitro bioassays were performed with HepG2/C3A human hepatocarcinoma cells (ATCC, CRL‐10741), obtained from the Rio de Janeiro Cell Bank (Brazil, code: BCRJ 0291). These cells correspond to a patented subclone of the human hepatocyte cell line HepG2/C3A (ATCC HB‐8065). The cells were cultured in DMEM medium (Dulbecco's modified Eagle's medium—Cultilab, Campinas, Brazil), supplemented with 10% fetal bovine serum, 0.1% antibiotic‐antimycotic solution (penicillin 10,000 IU/mL; streptomycin 10 mg/mL, Cultilab, Campinas, Brazil) and 0.3% of glucose (Merck, CAS 50‐99‐7). Cell culture was carried out in 25 cm^2^ ventilated flasks, kept at a controlled temperature of 37°C, in a 5.0% CO_2_ incubator with 80% humidity. When the cell culture reached 80% confluency, the cells were used in the cytotoxicity, genotoxicity, and potential mutagenicity assays.

### 2.3 Treatments

The tests were carried out with both types of effluent. HepG2/C3A cells were chosen based on OECD 487 guideline, which describes the in vitro mammalian cell micronucleus (MN) test [22]. Furthermore, this cell line has a metabolization system that includes phase I and II enzymatic activities, which can promote the activation and detoxification of DNA‐reactive chemicals [23]. Thus, this cell line is considered an efficient test system to be applied in the assessment of the cytotoxic and genotoxic potential of various substances [20].

The cells were exposed to different dilutions, as mentioned in item 2.1. For each type of test performed (cytotoxicity, genotoxicity, or mutagenicity), the appropriate negative (NC) and positive (PC) controls were used, as detailed in the description below. The incubator protected the cells from light during the exposure period.

### 2.4 Cytotoxicity Tests

To better characterize the effects of hair dyeing salon effluents on human cell lines, two methods based on different markers were used. The MTT test assesses cell viability through the mitochondrial metabolism of cells. It is a standard test recommended by international agencies. The Trypan Blue test assesses the integrity of the plasma membrane and allows for a faster assessment, generally used before a test to verify sublethal effects, such as the comet and MN tests [24, 25].

#### 2.4.1 Cell Viability (Trypan Blue Test)

The cell viability test with Trypan Blue dye (0.4%, Gibco, CAS 72‐57‐1) was performed according to the protocol described by Mani and Swargiary [26], with some modifications. Sterile 6‐well flat‐bottom plastic plates were used, and 5 × 10^5^ cells were seeded in each well with 2.0 mL of serum medium. Then, the plates were incubated under the same culture conditions mentioned above for a stabilization period of 24 h. After, the culture medium from the wells was removed to start the exposure. Then, according to the group evaluated, 400 μL of the sample was added individually to each well. The crude sample and the five dilutions of each effluent (DE‐1 to DE‐6/CE‐1 to CE‐6) were evaluated according to the ratio described in item 2.1 (20% of sample, 80% of culture medium). Only sterile culture medium without serum was used for NC, and Triton X‐100 solution (1%) (Vetec, CAS 9036‐19‐5) was used for PC in serum‐free culture medium. The exposure was performed in triplicate for 4 h.

After this time, the cells were removed by adding 200 μL of 0.5% trypsin (Sigma Aldrich, T4049), and the plate was kept in an incubator at 37°C for 5 min. Then, trypsin was inactivated with 600 μL of serum medium, and the cell suspension was transferred to microtubes, corresponding to each treatment. Then, in another microtube, 20 μL of Trypan Blue was mixed with 20 μL of the homogenized cell suspension; 40 μL of this mixture was transferred to a Neubauer chamber for counting live (unstained) and dead (blue) cells under light microscopy (×40 objective). The results of cell viability were transformed into percentages and compared with the NC results—viable concentrations were those with viability values ≥ 80%.

#### 2.4.2 MTT Test

The MTT test (Thiazolyl blue tetrazolium bromide—CAS 298‐93‐1, Sigma Aldrich) was developed according to the protocol established by Mosmann [27], with some modifications. In a 96‐well plate, a total of 2.34 × 10^4^ cells were seeded in each well with 100 μL of serum culture medium. Only culture medium was added to the blank wells. The plates were incubated for 24 h in an incubator at 37°C to stabilize the cells.

After, each well had its content removed and replaced by 200 μL of the sample. The crude sample and the five dilutions of each effluent (DE‐1 to DE‐6/CE‐1 to CE‐6) were evaluated, following the proportion described in item 2.1 (20% of sample: 80% of culture medium). In the wells destined for the NC, 200 μL of serum‐free culture medium was added, while in the wells destined for the PC, the same volume of Triton X‐100 solution (1%) was added (Vetec, CAS 9036‐19‐5). Each plate represented 10 replicates of each treatment. The exposure was carried out in an incubator for 24 h under the same conditions of cell culture.

After the exposure, the treatments were removed from the wells, and 150 μL of MTT solution (0.001 mg/mL) was added. The plate was incubated for 4 h at 37°C. Then, the MTT solution was discarded, and 100 μL of dimethyl sulfoxide (DMSO—Exodus, CAS 6768‐5) was added to each well. The absorbance of each well was read using a microplate reader (Infinite M200 PRO, TECAN, Switzerland) at 540 nm.

### 2.5 Genotoxicity and Mutagenicity Tests

#### 2.5.1 Dilutions

For the testing of genotoxicity and mutagenicity, HepG2/C3A cells were exposed only to dilutions that did not show cytotoxicity by Trypan Blue and MTT tests (≥ 80% viability), that is, DE‐4, DE‐5, DE‐6, CE‐4, CE‐5, CE‐6. The exposure period for genotoxicity and mutagenicity tests was established according to the guidance of OECD standard 487 [22], which indicates 3–6 h.

The cells were exposed in 25 cm^2^ culture flasks with 2.0 mL of the sample and 8.0 mL of culture medium, totaling 10.0 mL of final volume and maintaining the same ratio used in cytotoxicity tests.

#### 2.5.2 Comet Assay

The comet assay was performed to evaluate the genotoxic potential of the effluents, according to the protocol established by Singh et al. [28], Tice et al. [29], and Collins et al. [30], with some modifications. Initially, 5.0 × 10^5^ cells were added to each culture flask and kept for 24 h at 37°C under the same culture conditions to allow stabilization.

Subsequently, the culture medium was replaced with the different samples for an exposure of 4 h. The NC was performed with the solution of 5 μL/mL of PBS with culture medium, and the PC with the solution of 10 mM of methylmethane sulfonate (MMS, Sigma Aldrich, CAS 66‐27‐3). The assay was performed in triplicate.

After the exposure, the obtained cell suspensions were submitted to the cell viability test. Treatments with confirmed cell viability greater than 80% were used in the comet assay. In different cryovials, 20 μL of the cell suspension was mixed with 20 μL of Trypan Blue (0.4%), according to the methodology described by Salvadori, Ribeiro, and Fenech [31]. To obtain cell viability, 100 cells per treatment were quantified. The unstained cells were considered alive, and the blue cells were considered dead. Once cell viability was confirmed, 20 μL of the cell suspension was mixed with 120 μL of low‐melting‐point agarose (0.5%—37°C). These mixtures were placed on slides previously coated with normal agarose (1.5%) and immediately covered with a coverslip. For agarose solidification and coverslip removal, the slides were cooled at 4°C for 20 min. After removal of the coverslips, the slides were subjected to lysis solution (1.0 mL of Triton X‐100, 10.0 mL of DMSO, and 89.0 mL of stock lysis solution—2.5 M NaCl, 100 mM EDTA, 10 mM Tris, and ~8 g of solid NaOH to 1 L; pH 10.0), at 4°C for at least 1 h. After, the slides were transferred to an electrophoresis vat and covered with cold and freshly prepared alkaline buffer (300 mM NaOH + 1 mM EDTA; pH > 13.0) at 4°C for 20 min for DNA denaturation. Then, the slides were submitted to electrophoresis at 39.0 V (~0.8 V/cm) and 300 mA for 20 min. Immediately after electrophoresis, the slides were removed from the vat, neutralized with Tris buffer (0.4 M Trizma hydrochloride; pH 7.5), fixed in absolute ethanol for 10 min, and stored at 4°C.

For analysis, each slide received 50.0 μL of GelRed (15.0 μL of 10 000X GelRed in water, 5.0 mL of 1 M NaCl, and 45.0 mL of distilled water) and a new coverslip. The analyses were performed under an epifluorescence microscope (DM4000B, Leica, Wetzlar, Germany), using the B 34 filter (excitation: *λ* = 420–490 nm, barrier: *λ* = 520 nm) and the ×40 objective. DNA damage was determined considering the parameter of tail intensity [22], which represents the percentage of fragments of DNA in the tail of the comet carried throughout electrophoresis and observed by the Comet Assay IV software (Instem, UK).

#### 2.5.3 Cytokinesis‐Blockage MN Test

The evaluation of the possible mutagenic effects of the effluents was performed using the MN test with cytokinesis block, following the protocol described by OECD 487 [17, 30, 32], with some modifications. For the assay, 10^6^ cells were seeded in 25 cm^2^ culture flasks and kept for 24 h under culture conditions for cell stabilization. Then, the culture medium was removed and the cells were subjected to treatments for another 4 h. The NC was performed with the 5 μL/mL PBS solution with culture medium and the PC with the 10 mM methylmethane sulfonate solution (MMS, Sigma Aldrich, CAS 66‐27‐3). The assay was performed in triplicate.

At the end of this period, the treatments were removed, cultures were washed with PBS and submitted to a solution of cytochalasin B (3.5 μg/mL—Cayman Chemical Company, 14 930‐96‐2) in 5 mL of complete culture medium. The flasks remained incubated for another 30 h to obtain binucleated cells. Then, the cells were collected, treated with hypertonic sodium citrate solution (1%), and fixed using Carnoy fixative (ethanol: acetic acid, 3:1 v/v). To prepare the slides, the cells were centrifuged and then homogenized in 0.5 mL of Carnoy fixative. A few drops of the cell suspension were placed on slides at 4°C, covered with a water layer. After drying at room temperature, the slides were stained with 5% Giemsa solution for 8 min.

The analyses were performed under a light microscope (Leica DM500, ×100 objective). A total of 6000 binucleate cells were counted per treatment (1000 cells/slide, 2 slides/flask, 3 flasks/treatment). Among the total number of binucleate cells analyzed, normal binucleate cells and those with MN, nucleoplasmic bridges, and/or nuclear buds were counted separately. The criteria used for the analyses were those indicated by Fenech and Knasmüller [33]. Binucleate cells with intact cytoplasmic membranes and nuclear envelopes, nuclei of similar sizes and not overlapping, with the same staining pattern and intensity were counted.

Another evaluation performed with HepG2/C3A cell culture was the determination of the cytokinesis‐block proliferation index (CBPI). This index indicates the average number of cell cycles that the cells went through during the period of exposure to cytochalasin B. For each treatment, 1500 cells (500 cells/flask) were counted, as described in OECD guideline n. 487 [32]. The CBPI index was calculated according to Equation (1).

(1) $$\text{CBPI}=\frac{\left(M1\right)+\left(2\times M2\right)+\left(3\times M3\right)}{N}$$

where M1 is the cells with one nucleus; M2 is the cells with two nuclei; M3 is the cells with three nuclei or more; *N* is the total number of cells analyzed

Simultaneously with the MN test, the cytotoxicity of the effluents studied was evaluated by determining the cytotoxicity estimate (%*CytE*). This index indicates the percentage of inhibition of cell proliferation induced by the treatment after the exposure period. The determination of cytostatic activity was based on the previously described CBPI, according to Equation (2) [32].

(2) $$\%\text{CytE}=100-100\times \frac{\left({\text{CBPI}}_{t-1}\;\right)}{\left({\text{CBPI}}_{c-1}\right)}$$

where *CBPI*_t_ is the CBPI of treatment; *CBPI*_c_ is the CBPI of negative control.

Another analysis performed in this study was the replication index (RI), also described by Trompowsky et al. [34]. The RI indicates the relative number of cell cycles, per cell, during the exposure to cytochalasin B, comparing the results of the treatments with those of the NC. To determine RI, 500 cells per flask were analyzed for the incidence of binucleate cells and multinucleated cells. RI was calculated according to Equation (3) [32].

(3) $$\mathrm{RI}\;\left(\%\right)=\frac{\left[\left(\text{Number of binucleated cells}\right)+2\times \left(\text{number of multinucleated cells}\right)÷500\;\right]t}{\left[\left(\text{Number of binucleated cells}\right)+2\times \left(\text{number of multinucleated cells}\right)÷500\;\right]c\;}\times 100$$

where *t* is the treatment; *c* is the negative control.

### 2.6 Statistical Analysis

The statistical analyses of all tests were performed with the aid of the statistical software GraphPad Prism 9.0 (La Jolla, USA). All data obtained were evaluated using the D'Agostino and Pearson normality test. Subsequently, according to the result obtained in the above test, it was directed to either the parametric test (ANOVA/Dunnett) or the nonparametric test (Kruskal–Wallis/Dunn). The statistical analyses performed considered a confidence level of 95%.

## 3 Results

### 3.1 Cytotoxicity Tests

**FIGURE 1:**
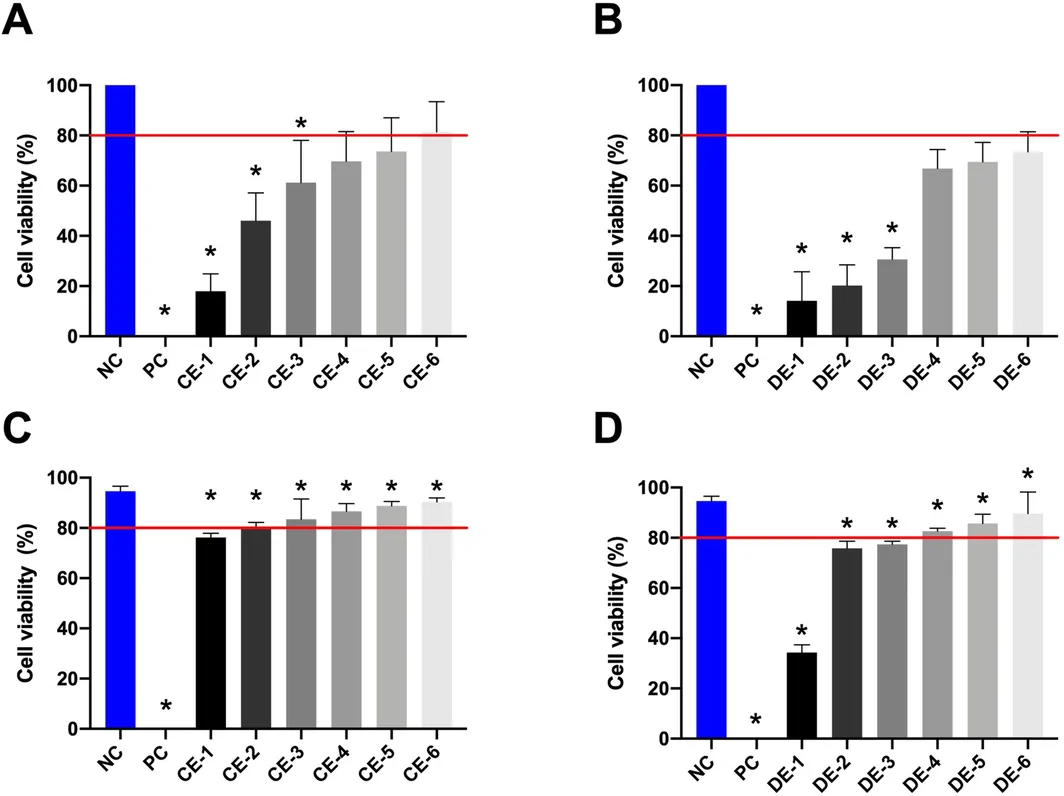
Cell viability evaluated by MTT and Trypan Blue tests on HepG2/C3A cells exposed to beauty salon effluents. Results are shown as means and standard deviation. (A, B) MTT test; (C, D) Trypan Blue test. CE‐1: raw sample (100%); CE‐2: sample diluted by 50.00%; CE‐3: sample diluted by 25.00%; CE‐4: sample diluted by 12.50%; CE‐5: sample diluted by 6.25%; CE‐6: sample diluted by 3.125%; DE‐1: raw sample (100%); DE‐2: diluted by 50.00%; DE‐3: sample diluted by 25.00%; DE‐4: sample diluted by 12.50%; DE‐5: sample diluted by 6.25%; DE‐6: sample diluted by 3.125%; NC: negative control; PC: positive control. The red line indicates the cytotoxicity threshold of 80%. *Significant when compared to NC (*p* < 0.05—Kruskal–Wallis/Dunn for (A, B); ANOVA/Dunnett for (C, D)).

The results of the MTT test, obtained with the different dilutions of the CE sample (Figure 1A) and the DE sample (Figure 1B), showed cytotoxicity only for treatments CE‐1, CE‐2, DE‐1, DE‐2, and DE‐3.

Regarding the Trypan Blue test, the results of the CE sample (Figure 1C) and the DE sample (Figure 1D) showed cytotoxicity only for the DE‐1 treatment. However, although the other treatments presented satisfactory cell viability (> 80%), there was a statistical difference for all treatments when compared to NC.

**FIGURE 2:**
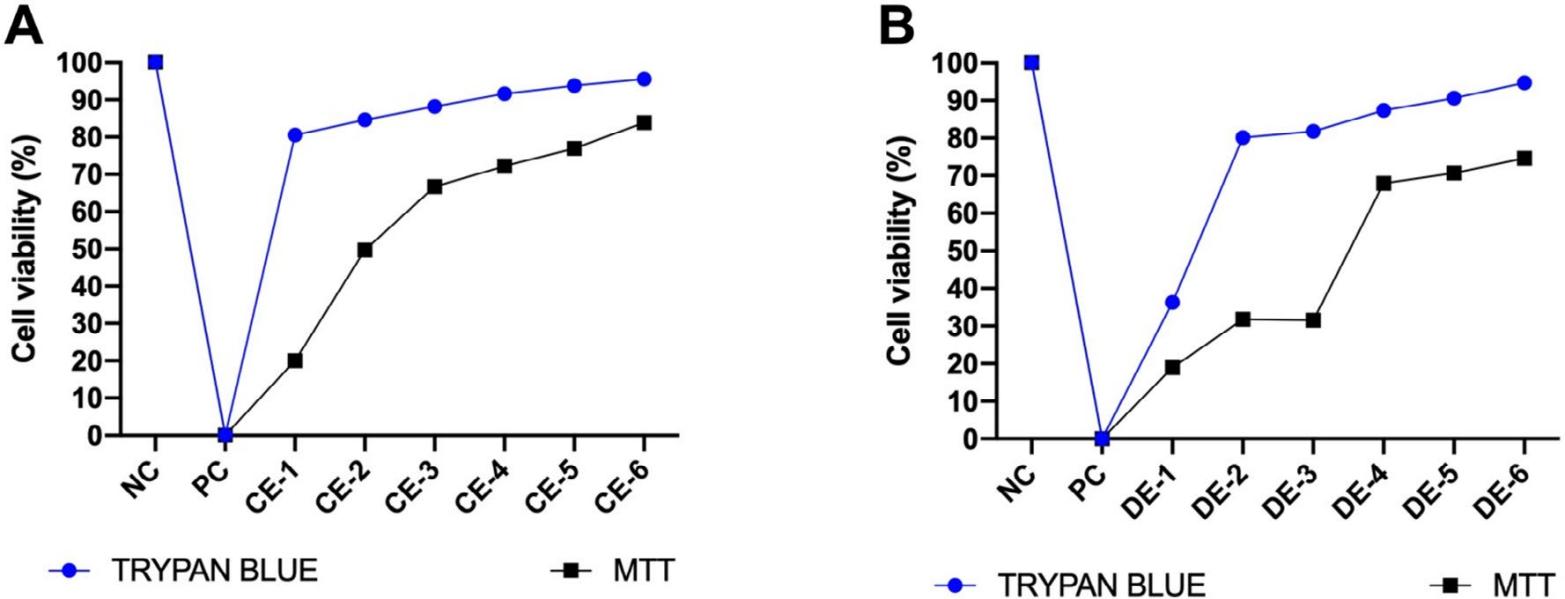
Comparison of the results obtained from the MTT and Trypan Blue tests performed with HepG2/C3A cells exposed to beauty salon effluents. (A) CE exposure; (B) DE exposure. CE‐1: raw sample (100%); CE‐2: sample diluted by 50.00%; CE‐3: sample diluted by 25.00%; CE‐4: sample diluted by 12.50%; CE‐5: sample diluted by 6.25%; CE‐6: sample diluted by 3.125%; DE‐1: raw sample (100%); DE‐2: diluted by 50.00%; DE‐3: sample diluted by 25.00%; DE‐4: sample diluted by 12.50%; DE‐5: sample diluted by 6.25%; DE‐6: sample diluted by 3.125%; NC: negative control; PC: positive control.

The results of the two cytotoxicity tests (MTT and Trypan Blue) were compared with each other, as shown in Figure 2. Both curves show relatively lower cell viability rates for the MTT test when compared with the Trypan Blue test. The curve pattern for CE was proportionally similar for both tests (Figure 2A), while there was a small difference for DE treatment (Figure 2B).

### 3.2 Comet Assay

The levels of primary DNA damage were evaluated by the comet assay by measuring the comet tail intensity (% of DNA). After the exposure, all dilutions of CE and DE (item 2.1) induced significant damage to HepG2/C3A cells (Table 1).

**TABLE 1:** —Results from the comet assay performed with HepG2/C3A cells exposed to beauty salon effluents.

	Treatment	Tail intensity (%)
CE	NC	5.37 ± 1.11
	PC	66.44 ± 2.02*
	CE‐4	35.00 ± 2.09* ^A,B^
	CE‐5	24.26 ± 2.44* ^A,C^
	CE‐6	14.33 ± 2.60^B,C^
DE	NC	4.98 ± 1.32
	PC	69.52 ± 4.53*
	DE‐4	15.07 ± 1.73* ^D,E^
	DE‐5	8.48 ± 1.61* ^D,F^
	DE‐6	5.10 ± 1.07^E,F^

*Note:* Results are shown as means and standard deviation.

Abbreviations: CE: complete effluent; CE‐4: sample diluted by 12.50%; CE‐5: sample diluted by 6.25%; CE‐6: sample diluted by 3.125%; DE: dye effluent; DE‐4: sample diluted by 12.50%; DE‐5: sample diluted by 6.25%; DE‐6: sample diluted by 3.125%; NC: negative control; PC: positive control.

* Significant when compared to NC.

Identical letters represent significant differences (*p* < 0.05—Kruskal–Wallis/Dunn).

### 3.3 MN Test

The genotoxic and the potential mutagenic effects of the different dilutions of CE and DE were evaluated by the MN test and all related endpoints. The frequencies of MN and nuclear buds did not show significant results when compared to NC (Table 2). No nucleoplasmic bridges were observed.

**TABLE 2:** —Results from the micronucleus test performed with HepG2/C3A cells exposed to beauty salon effluents.

Treatment	NB	MN	CBPI	CytE	RI
NC	0.20 ± 0.12	1.00 ± 0.22	1.59 ± 0.05	0.0%	100.0%
PC	1.70 ± 0.21*	4.20 ± 0.35*	1.24 ± 0.07*	61.0%	39.0%
CE‐4	0.40 ± 0.24	1.20 ± 0.20	1.31 ± 0.07*	47.0%	53.2%
CE‐5	0.40 ± 0.26	1.10 ± 0.17	1.35 ± 0.03*	41.2%	59.8%
CE‐6	0.20 ± 0.10	1.10 ± 0.28	1.40 ± 0.05*	32.1%	70.0%
DE‐4	0.50 ± 0.27	1.20 ± 0.10	1.36 ± 0.02*	38.2%	66.6%
DE‐5	0.30 ± 0.09	1.03 ± 0.16	1.43 ± 0.03*	26.2%	80.4%
DE‐6	0.00 ± 0.00	1.00 ± 0.12	1.44 ± 0.03*	26.1%	80.3%

*Note:* Results are shown as means and standard deviation.

Abbreviations: CBPI: cytokinesis‐block proliferation index; CE: complete effluent; CytE: cytotoxicity estimate; DE: dye effluent; MN: micronucleus; NB: nuclear bud; NC: negative control; PC: positive control; RI: replication index.

* Significant when compared to NC (*p* < 0.05—Kruskal–Wallis/Dunn).

The cytostatic effect was measured by the CBPI and showed that all dilutions tested of both CE and DE treatments were significantly different when compared to NC results.

## 4 Discussion

Water contamination by PCPs is growing exponentially and, consequently, has been causing considerable impacts on the environment and human health [35]. Among the effluents contaminated with PCPs are those derived from beauty salons. These effluents contain a complex content as they contain several chemical compounds, including highly toxic hair dyes [36] and products used for color fixation and hair washing.

The effluents generated in these procedures are continuously discharged into the sewage system, which often passes through treatment plants that are not prepared to deal with this type of waste. Thus, these residues can reach hydrological systems and bioaccumulate in organisms [37]. To assess the cytotoxicity of these effluents, which remain relatively unexplored in terms of their toxicity, both Trypan Blue and MTT tests were conducted.

From the results of the MTT test, it was possible to observe that the two highest concentrations of CE (CE‐1, CE‐2, and CE‐3) and the three highest concentrations of DE (DE‐1, DE‐2, and DE‐3) induced cell death by decreasing mitochondrial metabolism (cell viability less than 80%) (Figure 1A and 1B, respectively). Nevertheless, the Trypan Blue exclusion assay, which evaluates induction of cell death by loss of cell membrane integrity, showed all the concentrations (CE‐1, CE‐2, CE‐3, CE‐4, CE‐5, CE‐6, DE‐1, DE‐2, DE‐3, DE‐4, DE‐5, and DE‐6) to be cytotoxic to HepG2/C3A cells (Figure 1D). The cytotoxic effect of the effluents showed a dose‐dependent relationship, wherein the rise in cell viability was proportional to the decrease in the tested concentration. These results suggest greater impairment of mitochondrial activity compared to loss of plasma membrane integrity. However, both mitochondrial and plasma membrane impairments are noted, potentiating damage.

Three hypotheses can be suggested for the cytotoxicity. The first is based on reports that indicate apoptosis as a mitochondrial‐mediated process through lethal pro‐apoptotic components. These substances are trapped in the mitochondrial intermembranous space, being released into the cytosol through a protein structure called mitochondrial permeability transition pore (MPTP) [38, 39].

The opening of this pore can occur by different stimuli expansion of the mitochondrial matrix and/or entry of protons into this matrix; formation of reactive oxygen species; increase in intramitochondrial calcium; hydrophobic bile acids; and some drugs [39, 40]. In general, mammalian mitochondria work as central control points for some forms of apoptosis, making them the main target of the cell's signaling and survival system [39].

The second hypothesis is based on the toxic mechanism of action of some compounds, such as aromatic amines. Among them, it is important to highlight 1,4‐diamino‐2‐methylbenzene, a compound present in the brown hair dye that constituted the effluents. Nelms et al. [41] state that this aromatic amine can disrupt mitochondrial functions and cause cytotoxicity, as was confirmed by the MTT test applied in this study.

The third hypothesis refers to the suggestions published by Lui et al. [18], who describe the rupture of membranes as a consequence of necrosis during the final stages of apoptosis. Some authors describe this rupture as a possible consequence of apoptotic body formation, which occurs through the rearrangement of membranes during final apoptosis [42, 43, 44]. The consequent alteration in membrane permeability allows the entry of vital dyes, such as Trypan Blue.

Cytotoxicity tests are also used to define the concentration range to be applied in the genotoxicity, mutagenicity, or programmed cell death assays. Cell viability indicates the appropriate concentration to test a substance, ensuring that more than 80% of cells remain alive [45]. These tests also enable the assessment of how cells respond to the environmental conditions applied during the exposure, which is crucial to ensure that the concentration employed is nonlethal to the cells. It allows the verification of whether the substance has genotoxic potential and whether it interferes with the pace of the cell cycle, thereby altering the growth curve through delays and/or induction of apoptosis [46].

Studies show that MTT can overestimate or underestimate cell viability, especially in conditions that affect mitochondrial metabolism, while Trypan Blue offers a more direct estimate of cell viability with less risk of misinterpretations due to metabolic interferences [47, 48]. Additionally, Trypan Blue has been considered more reliable in direct comparisons with MTT, highlighting its superiority when cell viability needs to be precisely assessed to choose concentrations for further genetic toxicity studies [49]. In this context, Trypan Blue results were selected, as they revealed cell viability greater than 80% for the three highest dilutions of both samples (CE‐4, CE‐5, CE‐6, DE‐4, DE‐5, and DE‐6).

The genotoxicity evaluation performed by the comet assay revealed that all concentrations of CE and two of DE (DE‐4 and DE‐5) induced DNA fragmentation in HepG2/C3A cells, an effect evidenced by the increased intensity of the comet tail (Table 1). DNA fragmentation can be interpreted as indicative of an apoptotic process since the cells exhibit disruptions in the genetic material caused by the activation of endonucleases [50, 51].

The genotoxicity results obtained in the comet assay with HepG2/C3A cells exposed to CE and DE effluents, in conjunction with the previously described cytotoxicity results, showed a concentration‐dependent relationship, that is, the higher the concentration, the more pronounced the harmful effects. Nevertheless, CE exhibited a greater genotoxic potential than cytotoxic potential when compared to DE. These results indicate that the effects of CE are more closely associated with inducing damage to DNA structure rather than affecting plasma membranes and mitochondrial activities.

Based on the described findings, it can be concluded that more intricate effluents, such as those including the combination of shampoo, conditioner, and hair dye (CE), exhibited a higher genotoxic potential for HepG2/C3A cells compared to effluents containing only residues of these dyes (DE). These results provide crucial and unprecedented insights into effluents from beauty salons, suggesting increased toxicity of hair dyes when they are associated with surfactant compounds.

Considering the possible release of the components of these effluents into the environment, their impact could be even worse. Multiple compounds can be found simultaneously in aquatic environments, forming complex mixtures [52]. These mixtures can allow chemical interactions that alter the known mechanism of action for isolated compounds [53], triggering different toxic effects on the same test organism [52, 54].

The presence of dyes in effluents, regardless of quantity, is highly undesirable as they cause severe damage to aquatic ecosystems. The discharge of these effluents into water bodies leads to a decrease in dissolved oxygen levels and reduces the reoxygenation capacity. In addition to the negative esthetic effect, the presence of dyes also impairs photosynthesis by limiting light penetration into the water [55, 56].

However, the contamination scenario may differ when these dyes interact with natural aquatic organic matter, which contains various active substances, such as fulvic and humic acids. These acids, for instance, are known to complex metal ions and organic pollutants, like certain hair dyes, thereby increasing their overall adsorption capacity [57].

Aquatic environments can contain organic matter either dissolved in water or in particulate form, originating from both natural and anthropogenic sources. Dissolved organic matter (DOM) is composed of smaller molecules that pass through a filter, while particulate organic matter (POM) consists of larger particles that can be retained by filtration—typically those larger than 0.45 μm [58]. According to the same authors, studies report that the dynamic exchange between DOM and POM is a critical activity influencing the mobility and bioavailability of pollutants [58]. This is corroborated by Luo et al. [59], who state that the bioavailability, fate, and behavior of organic chemical substances in aquatic ecosystems are directly influenced by DOM and POM.

Pinheiro et al. [60] describe that DOM interacts with environmental contaminants through a wide range of mechanisms, including ion exchange, hydrogen bonding, charge transfer, covalent bonds, hydrophobic adsorption, and partition, which can affect the distribution, bioconcentration, and toxicity of these compounds in water. This information is reinforced by Zermane et al. [61], who demonstrated the binding of the dye Basic Yellow 28 to humic acids. Although this is not a hair dye, it verifies that this category of environmental contaminants is subject to interactions with DOM.

Based on our current knowledge, there are no studies that point to an interaction between hair dyes and the organic matter present in aquatic environments. However, according to Delgado‐Moreno et al. [62], hydrophobic organic contaminants are closely related to bioaccumulation and biomagnification processes. Thus, the presence of DOM in aquatic environments enables the sorption of hydrophobic organic contaminants, such as cationic hair dyes, thereby reducing their toxicity and bioaccumulation [63].

While numerous studies emphasize the necessity for research addressing the toxicity of chemical mixtures for conducting risk assessments [64], there remains a dearth of information regarding both the mechanism of action and the effects of mixtures of compounds employed in the hair coloring process [65]. The mechanisms underlying these interactions are not yet fully understood, demanding further investigation.

In general, genotoxicity and mutagenicity assays are employed to analyze the potential effects of complex mixtures without requiring precise chemical characterization of their components [66]. Whereas physicochemical analyses are insufficient to determine the effects of a mixture [67, 68], toxicogenetic tests are essential to allow the evaluation of its environmental risk potential. Another inference regarding the cytotoxic and genotoxic effects of the evaluated dye could be related to the toxic action of titanium dioxide (TiO_2_), one of the components of the hair dye studied (Table S1). As discussed in another manuscript published by our research group [69], which assessed the same effluents in vivo, TiO_2_ is found in the hair dye and could be responsible for impairing the test organisms. TiO_2_ particles can disrupt plasma membranes, inhibit mitochondrial activity, and even alter the functionality of lysosomes [42, 70]. In addition, TiO_2_ particles also induced cytotoxic damage to the hepatic cells of rodents [71, 72, 73, 74] and humans [75], also in a dose‐dependent manner.

Based on in vitro assays, Zhu et al. [76] proposed the mechanism by which TiO_2_ nanoparticles interact with and break DNA. The results of infrared spectroscopy showed that these nanoparticles bind to DNA in the phosphate group (P═O), forming P–O–Ti and loosening the C–O–P bond, without altering the nitrogen bases of the DNA. This alteration causes DNA breakage [42, 77], which explains the genetic material fragmentation as observed in this study by the comet assay. Additionally, TiO_2_ nanoparticles can induce apoptosis and necrosis in mammalian cells [18, 42].

Other authors, such as Galiotte et al. [78], also found a higher frequency of DNA breaks in hairdressers exposed to hair dyes when compared to a nonexposed control group. Considering the OECD guideline n. 489 [22], these breaks in DNA can be repaired, resulting in a nonpersistent effect, or be fixed in a mutation, resulting in a permanent change. In general, a mutagenic substance induces DNA damage, which is transmissible from the mother cell to the daughter cells [79]. Moreover, the review work published by Roberto and Christofoletti [80] reports various ecotoxic effects of TiO_2_ nanoparticles. This is also supported by another study developed by our research group, demonstrating the toxic impact of these effluents on aquatic organisms [69].

Another test also used in genotoxicity evaluations of substances and environmental samples (such as the effluents evaluated here) is the MN test. This test is performed to detect chromosome fragments, acentric chromosomes, and loss of whole chromosomes [31, 33, 81]. The results of the MN test presented here indicate the absence of micronuclei formation following the exposure of HepG2/C3A cells to all concentrations of the assessed effluents (CE and DE)

According to Houtgraaf et al. [82], cell cycle checkpoints are activated in response to genetic material damage, leading to a halt in the cycle. This pause allows the repair system to act on the DNA. When the repair occurs correctly, the cells continue their normal cell cycle without causing damage or alterations to daughter cells [83] Considering the description of both works cited above [82, 83] and based on the results of the present study, it can be inferred that the components of the evaluated effluents induced damage to the genetic material of HepG2/C3A cells, as observed by the comet assay. However, these damages must have undergone repair without being fixed in the daughter cells, which justifies the absence of alterations in the MN test, as established by Fenech [33].

In this study, additional cytotoxicity assessments were conducted concurrently with the MN test, including the CBPI, RI, and percentage of cytotoxicity estimate. The CBPI reveals the average number of cycles undergone by each cell during the cytochalasin B exposure period and can also be used to determine the levels of cell proliferation through the cytotoxicity estimate. Another recommended measurement is the RI, which directly indicates the extent of cell replication in the treated cultures relative to the data from the control test [84].

Cytotoxicity of an agent may result in cytostasis or cell death. Cytostasis is a state of temporary disruption of the cell cycle, in which cells stop actively dividing as a response to various stimuli or conditions in the cellular environment. This event is usually induced by the action of cell inhibitors, but it can also be induced by toxic agents that interfere with specific cell cycle pathways, causing their arrest. Since cytostasis can also be related to cell death, this component of cytotoxicity may be predominant and therefore should always be considered in the assessment of overall cytotoxicity. According to some authors, when the in vitro MN test is performed with cytochalasin B, it is recommended to apply two complementary cytotoxicity measurement methods, the CBPI and the RI [84, 85].

In this study, cultures exposed to effluents from beauty salons showed a significant decrease in CBPI and RI, as well as an increase in cytotoxicity estimate (Table 2). Decreased RI percentages were observed for CE and DE treatments and were associated with increased cytotoxicity estimates. The lowest RI rate was recorded for cells exposed to CE‐4 (RI = 53.2%; CA = 47.0%). Based on these results, it can be inferred that the high cytotoxicity estimate observed may be associated with cell cycle arrest, activated in response to prevent damage consolidation. This mechanism allows the repair of genetic material to occur over an extended period. This statement is justified by the presence of breaks in the genetic material, observed by the comet assay, and the absence of NM in the corresponding test. This process of cell cycle arrest may contribute to the apoptotic process and subsequent damage to the plasma membrane and/or mitochondria, as previously elucidated [75].

The CE and DE effluents showed cytotoxic and genotoxic effects on HepG2/C3A cells. There was DNA fragmentation, a decrease in CBPI and RI, and an increase in CA. These results are worrisome, as CE and DE effluents interfered with mitochondrial activity, cell membrane permeability and integrity, and cell cycle development. The results related to the effects observed in the cell cycle classify the effluents tested as toxic for the model employed.

## 5 Conclusion

Based on the results obtained in this study, the tested concentrations of CE and DE induced cytotoxic and genotoxic effects in human hepatocarcinoma (HepG2/C3A) cells. Although no significant frequencies of MN were observed—suggesting that the genotoxic damage was not transmitted to or fixed in daughter cells—the reduction in CBPI and RI, along with the increase in CA, indicates that these effluents pose risks to both environmental integrity and human health. These findings underscore the urgent need to establish strategies to mitigate their impacts and contribute valuable data on the toxicity of hair dye components and the residues generated during the dyeing process.

Although HepG2/C3A cells proved sensitive for evaluating the cytotoxic, genotoxic, and mutagenic potential of CE and DE, the results cannot be directly extrapolated to in vivo organisms. Therefore, further complementary studies with different bioindicators and endpoints are required to more accurately determine the effects of this contamination under realistic conditions. This demand is reinforced by the increasing global use of hair dyes, their complex and toxic composition, and their high potential for reactivity with other dyeing agents and environmental chemicals. In conclusion, the widespread use of hair dyes represents a relevant concern that may jeopardize both human health and environmental safety.

Thus, this study is directly aligned with the objectives of Sustainable Development Goal 6 (Clean Water and Sanitation), which emphasizes the need to ensure the availability and sustainable management of water and sanitation for all. Within this context, the mitigation of hair dye pollutants requires actions focused on water depollution, sanitation, disinfection, and wastewater treatment, as well as the development of specialized monitoring systems to detect and control emerging contaminants in aquatic environments.

## Author Contributions

**Letícia Cristina Gonçalves:** conceptualization, methodology, investigation, formal analysis, data curation, writing – original draft preparation. **Matheus Mantuanelli Roberto:** conceptualization, formal analysis, investigation, writing – review and editing, supervision. **Adriana Fabiana Corrêa da Silva:** methodology, investigation. **Maria Aparecida Marin‐Morales:** conceptualization, writing – review and editing, supervision, project administration, resources, funding acquisition.

## Funding

This work was supported by the Conselho Nacional de Desenvolvimento Científico e Tecnológico, Project PP (n306062/2019‐4).

## Conflicts of Interest

The authors declare no conflicts of interest.

## Supporting information


**Table S1:** Statistical data related to the cell viability assessment (Figure 1—MTT and Trypan Blue tests) on HepG2/C3A cells exposed to beauty salon effluents.

## Data Availability

The data that support the findings of this study are available from the corresponding author upon reasonable request.

## References

[1] R. A. G. Oliveira , T. B. Zanoni , G. G. Bessegato , D. P. Oliveira , G. A. Umbuzeiro , and M. V. B. Zanoni , “Chemistry and Toxicity of Hair Dyes,” Química Nova 37, no. 6 (2014): 1037–1046, 10.5935/0100-4042.20140143.

[2] K. Hanumanthayya , G. Balasubrahmanyam , M. Mohan , S. Mohan , and N. Nikhil , “Hair Coloring—Hair Dyeing,” RGUHS Journal of Medical Sciences 8, no. 1 (2018): 13–17, 10.26463/rjms.8_1_11.

[3] E. D. Melo and A. H. Mounteer , “Evaluation of the Treatability of Effluents From Hair Cosmetics Industries by Aerobic Biological Processes,” Augustus Magazine 24, no. 49 (2019): 155–178, 10.15202/1981896.2019v24n49p155.

[4] K. G. Pavithra , P. S. Kumar , V. Jaikumar , and P. S. Rajan , “Removal of Colorants From Wastewater: Sources and Treatment Strategies,” Journal of Industrial and Engineering Chemistry 75 (2019): 1–19, 10.1016/j.jiec.2019.02.011.

[5] S. Maifadi , S. D. Mhlanga , E. N. Nxumalo , M. M. Motsa , and A. T. Kuvarega , “Analysis and Pretreatment of Beauty Hair Salon Wastewater Using a Rapid Granular Multimedia Filtration System,” Journal of Water Process Engineering 33 (2020): 101050, 10.1016/j.jwpe.2019.101050.

[6] E. Brillas and C. A. Martinez‐Huitle , “Decontamination of Wastewater Containing Synthetic Organic Dyes by Electrochemical Methods: An Update Review,” Applied Catalysis B: Environmental 166‐167 (2015): 603–643, 10.1016/j.apcatb.2014.11.016.

[7] J. Xu , D. Wei , F. Wang , C. Bai , and Y. Du , “Bioassay: A Useful Tool for Evaluating Reclaimed Water Safety,” Journal of Environmental Sciences 88 (2020): 165–176, 10.1016/j.jes.2019.08.014. 31862058

[8] M. A. Nkansah , F. Opoku , J. H. Ephraim , D. D. Wemegah , and L. M. Tetteh , “Characterization of Beauty Salon Wastewater From Kwame Nkrumah University of Science and Technology, Kumasi, Ghana, and Its Surrounding Communities,” Environmental Health Insights 10 (2016): 1–9, 10.1177/EHI.S40360. PMC5004995 27594788

[9] V. A. Sousa , F. D. A. Sousa , A. E. F. Marques , and B. A. A. Moreira , “Cosmetic Toxicology: Assessment of the Risk That Healthy Products Bring to Health,” Visão Acadêmica 20, no. 4 (2019): 78–93.

[10] J. M. Brausch and G. M. Rand , “A Review of Personal Care Products in the Aquatic Environment: Environmental Concentrations and Toxicity,” Chemosphere 82, no. 11 (2011): 1518–1531, 10.1016/j.chemosphere.2010.11.018. 21185057

[11] D. Montes‐Grajales , M. Fennix‐Agudelo , and W. Miranda‐Castro , “Occurrence of Personal Care Products as Emerging Chemicals of Concern in Water Resources: A Review,” Science of the Total Environment 595 (2017): 601–614, 10.1016/j.scitotenv.2017.03.286. 28399499

[12] E. S. Machado , G. V. F. C. Silva , L. M. C. Moraes , M. B. Marques , R. B. Marques , and L. D. Silva , “Toxicological Aspects Related to the Use of Cosmetics in Hair Conservation, Straightening and Dyeing: A Literature Review,” Intertox Journal of Toxicology, Environment and Risk Society 10, no. 1 (2017): 5–18, 10.22280/revintervol10ed1.270.

[13] I. G. Portis , F. R. G. Figueiredo , R. V. Pena , et al., “Cytogenetic Bioassay for the Characterization of Mutagenicity and Cytotoxicity of *Choclhospermum regium* ,” Electronic Journal of the Evangelical Faculty, CERES 5, no. 1 (2016), 10.36607/refacer.v5i1.3357.

[14] N. Ourahmoune and H. E. Jurdi , “Beauty Salon—A Marketplace Icon,” Consumption Markets & Culture 24, no. 6 (2020): 611–619, 10.1080/10253866.2020.1741356.

[15] R. Eggen , J. Hollender , A. Joss , M. Schärer , and C. Stamm , “Reducing the Discharge of Micropollutants Into the Aquatic Environment: The Benefits of Modernising Wastewater Treatment Plants,” Environmental Science & Technology 48, no. 14 (2014): 7683–7689, 10.1021/es500907n. 24915506

[16] S. O. Rogero , A. B. Lugão , T. I. Ikeda , and A. S. Cruz , “In Vitro Cytotoxicity Test: A Comparative Study Between Two Methodologies,” Materials Research 6, no. 3 (2003): 317–320, 10.1590/S1516-14392003000300003.

[17] A. T. Natarajan and F. Darroudi , “Use of Human Hepatoma Cells for In Vitro Metabolic Activation of Chemical Mutagens/Carcinogens,” Mutagenesis 6, no. 5 (1991): 399–403, 10.1093/mutage/6.5.399. 1665540

[18] Q. Liu , “Pollution and Treatment of Dye Wastewater,” IOP Conference Series: Earth and Environmental Science 514 (2020): 052001, 10.1088/1755-1315/514/5/052001. PMC7350608 34191946

[19] G. F. Vieira , C. C. Santos , R. S. Figueiredo , et al., “Electro‐Oxidation of Wastewater From a Beauty Salon: The Influence of Electrolyte Type in the Removal of Organic Load and Energy Consumption,” Process Safety and Environment Protection 177 (2023): 1260–1271, 10.1016/j.psep.2023.07.078.

[20] L. R. D. Sommaggio , D. E. C. Mazzeo , J. A. Malvestiti , R. F. Dantas , and M. A. Marin‐Morales , “Influence of Ozonation and UV/H2O2 on the Genotoxicity of Secondary Wastewater Effluents,” Science of the Total Environment 919 (2024): 170883, 10.1016/j.scitotenv.2024.170883. 38354810

[21] G. Reifferscheid , C. Ziemann , D. Fieblinger , et al., “Measurement of Genotoxicity in Wastewater Samples With the In Vitro Micronucleus Test: Results of a Round‐Robin Study in the Context of Standardisation According to ISO,” Mutation Research, Genetic Toxicology and Environmental Mutagenesis 649, no. 1–2 (2008): 15–27, 10.1016/j.mrgentox.2007.07.015. 17980648

[22] Organisation for Economic Co‐Operation and Development (OECD) , Test Guideline No. 489: In Vivo Mammalian Alkaline Comet Assay. OECD Guidelines for the Testing of Chemicals (OECD Publishing, 2016).

[23] G. V. Crippa , T. A. Zanetti , B. I. Biazi , et al., “Up and Down‐Regulation of mRNA in the Cytotoxicity and Genotoxicity of Plumbagin in HepG2/C3A,” Environmental Toxicology and Pharmacology 75 (2020): 103328, 10.1016/j.etap.2020.103328. 32000057

[24] A. Kimura , A. Miyata , and M. Honma , “A Combination of In Vitro Comet Assay and Micronucleus Test Using Human Lymphoblastoid TK6 Cells,” Mutagenesis 28, no. 5 (2013): 583–590, 10.1093/mutage/get036. 23863314

[25] B. A. Avelar‐Freitas , V. G. Almeida , M. C. X. Pinto , et al., “Trypan Blue Exclusion Assay by Flow Cytometry,” Brazilian Journal of Medical and Biological Research 47 (2014): 307–315, 10.1590/1414-431X20143437. 24652322 PMC4075294

[26] S. Mani , M. Singh , A. Kumar , “In Vitro Cytotoxicity Analysis: MTT/XTT, Trypan Blue Exclusion,” in Animal Cell Culture: Principles and Practice, ed. Alexander E. Kalyuzhny (Springer International Publishing, 2023), 267–284.

[27] T. Mosmann , “Rapid Colorimetric Assay for Cellular Growth and Survival: Application to Proliferation and Cytotoxicity Assays,” Journal of Immunological Methods 65, no. 1–2 (1983): 55–63, 10.1016/0022-1759(83)90303-4. 6606682

[28] N. P. Singh , M. T. Mccoy , R. R. Tice , and E. L. Schneider , “A Simple Technique for Quantitation of Low Levels of DNA Damage in Individual Cells,” Experimental Cell Research 175, no. 1 (1988): 184–191, 10.1016/0014-4827(88)90265-0. 3345800

[29] R. R. Tice , E. Agurell , D. Anderson , et al., “Single Cell Gel/Comet Assay: Guidelines for In Vitro and In Vivo Genetic Toxicology Testing,” Environmental and Molecular Mutagenesis 35, no. 3 (2000): 206–221, 10.1002/(SICI)1098-2280(2000)35:3<206::AID-EM8>3.0.CO;2-J. 10737956

[30] A. R. Collins , “The Comet Assay for DNA Damage and Repair: Principles, Applications, and Limitations,” Molecular Biotechnology 26, no. 3 (2004): 249–261, 10.1385/MB:26:3:249. 15004294

[31] D. M. F. Salvadori , L. R. Ribeiro , and M. Fenech , “Teste do micronúcleo em células humanas In vitro,” in Mutagênese ambiental, ed. L. R. Ribeiro , D. M. F. Salvadori , and E. K. Marques (ULBRA, 2003), 201–223.

[32] The Organisation for Economic Co‐Operation and Development (OECD) , Test No. 487. Vitro Mammalian Cell Micronucleus Test. OECD Guidelines for the Testing of Chemicals (OECD Publishing, 2023), https://www.oecd‐ilibrary.org/docserver/9789264264861‐en.pdf?expires=1732802819&id=id&accname=guest&checksum=0BEF0AB9D1BEFF72DBF5CB967E66F3F6.

[33] M. Fenech and S. Knasmüller , The Micronucleus Assay in Toxicology (Royal Society of Chemistry, 2019).

[34] A. C. M. Trompowsky Von , T. R. Conde , R. C. Lemos , et al., “In Vitro Genotoxicity of Nitroimidazoles as a Tool in the Search of New Trypanocidal Agents,” Memórias do Instituto Oswaldo Cruz 114 (2019): 190017, 10.1590/0074-02760190017. PMC6598759 31271593

[35] J. P. P. Lima , E. D. Melo , and A. Aguiar , “Characteristics and Ways of Treating Cosmetic Wastewater Generated by Brazilian Industries: A Review,” Process Safety and Environment Protection 168 (2022): 601–612, 10.1016/j.psep.2022.10.031.

[36] K. Sampathkumar and S. Yesudas , “Hair Dye Poisoning and the Developing World,” Journal of Emergencies, Trauma, and Shock 2, no. 2 (2009): 129–131, 10.4103/0974-2700.50749. 19561974 PMC2700586

[37] L. Qin , H.‐Y. Deng , S.‐J. Chen , and W. Wei , “Meta‐Analysis on the Relationship Between Hair Dye and the Incidence of Non‐Hodgkin's Lymphoma,” Medical Principles and Practice 28, no. 3 (2019): 222–230, 10.1159/000496447. 30583293 PMC6597908

[38] A. P. Halestrap , G. P. Mcstay , and S. J. Clarke , “The Permeability Transition Pore Complex: Another View,” Biochimie 84, no. 2–3 (2002): 153–166, 10.1016/S0300-9084(02)01375-5. 12022946

[39] G. Morciano , N. Naumova , P. Koprowski , et al., “The Mitochondrial Permeability Transition Pore: An Evolving Concept Critical for Cell Life and Death,” Biological Reviews 96, no. 6 (2021): 2489–2521, 10.1111/brv.12764. 34155777

[40] S. Nesci , “The Mitochondrial Permeability Transition Pore in Cell Death: A Promising Drug Binding Bioarchitecture,” Medicinal Research Reviews 40, no. 2 (2020): 811–817, 10.1002/med.21635. 31617227

[41] M. D. Nelms , G. Ates , J. C. Madden , et al., “Proposal of an *in Silico* Profiler for Categorisation of Repeat Dose Toxicity Data of Hair Dyes,” Archives of Toxicology 89, no. 5 (2015): 733–741, 10.1007/s00204-014-1277-8. 24888375

[42] R. Mohammadinejad , M. A. Moosavi , S. Tavakol , et al., “Necrotic, Apoptotic and Autophagic Cell Fates Triggered by Nanoparticles,” Autophagy 15 (2019): 4–33, 10.1080/15548627.2018.1509171. 30160607 PMC6287681

[43] O. V. Saik , V. V. Nimaev , D. B. Usmonov , et al., “Prioritization of Genes Involved in Endothelial Cell Apoptosis by 46 Their Implication in Lymphedema Using an Analysis of Associative Gene Networks With ANDSystem,” BMC Medical Genomics 12 (2019): 47, 10.1186/s12920-019-0492-9. 30871556 PMC6417156

[44] J. Qualeri , C. Cevallos , and M. V. Delpino , “Apoptosis in Infectious Diseases as a Mechanism of Immune Evasion and Survival,” Advances in Protein Chemistry and Structural Biology 125 (2021): 1–24, 10.1016/bs.apcsb.2021.01.001. 33931136

[45] L. Tolosa , M. T. Donato , and J. Gómez‐Lechón , “General Cytotoxicity Assessment by Means of the MTT Assay,” in Methods in Molecular Biology, ed. Mathieu Vinken and Vera Rogiers (Springer, 2014), 333–348, 10.1007/978-1-4939-2074-7_26. 26272156

[46] H. Turkez , M. R. Arslan , and O. Ozdemir , “Genotoxicity Testing: Progress and Prospects for the Next Decade,” Expert Opinion on Drug Metabolism & Toxicology 13, no. 10 (2017): 1089–1098, 10.1080/17425255.2017.1375097. 28889778

[47] A. A. Stepanenko and V. V. Dmitrenko , “Pitfalls of the MTT Assay: Direct and Off‐Target Effects of Inhibitors Can Result in Over/Underestimation of Cell Viability,” Gene 574, no. 2 (2015): 193–203, 10.1016/j.gene.2015.08.009. 26260013

[48] D. M. Chung , J. H. Kim , and J. K. Kim , “Evaluation of MTT and Trypan Blue Assays for Radiation‐Induced Cell Viability Test in HepG2 Cells,” International Journal of Radiation Research 13, no. 4 (2015): 331, 10.7508/ijrr.2015.04.006.

[49] D. S. Masson‐Meyers , V. V. Bumah , and C. S. Enwemeka , “A Comparison of Four Methods for Determining Viability in Human Dermal Fibroblasts Irradiated With Blue Light,” Journal of Pharmacological and Toxicological Methods 79 (2016): 15–22, 10.1016/j.vascn.2016.01.001. 26780674

[50] X. Zhai , J. Lu , Y. Wang , F. Fang , B. Li , and W. Gu , “Reversal Effect of Bufalin on Multidrug Resistance in K562/VCR Vincristine‐Resistant Leukemia Cell Line,” Journal of Traditional Chinese Medicine 34, no. 6 (2014): 678–683, 10.1016/S0254-6272(15)30082-0. 25618972

[51] W. M. Kuwabara , R. Curi , and T. C. Alba‐Loureiro , “Autophagy Is Impaired in Neutrophils From Streptozotocin‐Induced Diabetic Rats,” Frontiers in Immunology 8 (2017): 24, 10.3389/fimmu.2017.00024. 28163707 PMC5247474

[52] C. L. S. Sisinno and E. C. Oliveira‐Filho , Princípios de Toxicologia Ambiental, 1st ed. (Interciência, 2021), 198.

[53] E. Geiger , R. Hornet‐Gausterer , and M. T. Saçan , “Single and Mixture Toxicity of Pharmaceuticals and Chlorophenols to Freshwater Algae *Chlorella vulgaris* ,” Ecotoxicology and Environmental Safety 129 (2016): 189–198, 10.1016/j.ecoenv.2016.03.032. 27045919

[54] M. E. Varona‐Uribe , C. H. Torres‐Rey , S. Díaz‐Criollo , et al., “Exposure to Pesticide Mixtures and DNA Damage Among Rice Field Workers,” Archives of Environmental & Occupational Health 71, no. 1 (2016): 3–9, 10.1080/19338244.2014.910489. 24972111

[55] S. Yao , M. Fabbricino , L. Pontoni , et al., “Characterization of Anthropogenic Organic Matter and Its Interaction With Direct Yellow 27 in Wastewater: Experimental Results and Perspectives of Resource Recovery,” Chemosphere 286, no. 1 (2022): 131528, 10.1016/j.chemosphere.2021.131528. 34303051

[56] M. Jawaduddin , Z. Su , M. S. Siddique , S. Rashid , and W. Yu , “Purifying Surface Water Contaminated With Azo Dyes Using Nanofiltration: Interactions Between Dyes and Dissolved Organic Matter,” Chemosphere 361 (2024): 142438, 10.1016/j.chemosphere.2024.142438. 38797203

[57] Y. Fan , C. Zheng , H. Liu , C. He , Z. Shen , and T. C. Zhang , “Effect of pH on the Adsorption of Arsenic(V) and Antimony(V) by the Black Soil in Three Systems: Performance and Mechanism,” Ecotoxicology and Environmental Safety 191 (2020): 110145, 10.1016/j.ecoenv.2019.110145. 31954214

[58] B. Hu , P. Wang , C. Wang , and T. Bao , “Photogeochemistry of Particulate Organic Matter in Aquatic Systems: A Review,” Science of the Total Environment 806, no. 3 (2022): 150467, 10.1016/j.scitotenv.2021.150467. 34592285

[59] J. Luo , M. Ma , C. Liu , J. Zha , and Z. Wang , “Impacts of Particulate Organic Carbon and Dissolved Organic Carbon on Removal of Polycyclic Aromatic Hydrocarbons, Organochlorine Pesticides, and Nonylphenols in a Wetland,” Journal of Soils and Sediments 9 (2009): 180–187, 10.1007/s11368-009-0081-1.

[60] J. P. S. Pinheiro , F. M. Windsor , R. W. Wilson , and C. R. Tyler , “Global Variation in Freshwater Physico‐Chemistry and Its Influence on Chemical Toxicity in Aquatic Wildlife,” Biological Reviews 96 (2021): 1528–1546, 10.1111/brv.12711. 33942490

[61] F. Zermane , B. Cheknane , J. P. Basly , O. Bouras , and M. Baudu , “Influence of Humic Acids on the Adsorption of Basic Yellow 28 Dye Onto an Iron Organo–Inorgano Pillared Clay and Two Hydrous Ferric Oxides,” Journal of Colloid and Interface Science 395 (2013): 212–216, 10.1016/j.jcis.2012.12.038. 23332940

[62] L. Delgado‐Moreno , L. Wu , and J. Gan , “Application of Isotope Dilution Method for Measuring Bioavailability of Organic Contaminants Sorbed to Dissolved Organic Matter (DOM),” Aquatic Toxicology 165 (2015): 129–135, 10.1016/j.aquatox.2015.05.006. 26037097

[63] Y. Huang , Y. Tian , L. Xie , et al., “The Application of Two‐Dimensional Correlation Spectroscopy for the Binding Properties of Heavy Metals Onto Digestate‐Derived DOM From Anaerobic Digestion of Chicken Manure,” Ecotoxicology and Environmental Safety 204 (2020): 111129, 10.1016/j.ecoenv.2020.111129. 32805505

[64] E. J. Calabrese , “Toxicological Consequences of Multiple Chemical Interactions: A Primer,” Toxicology 105 (1995): 121–135, 10.1016/0300-483X(95)03206-U. 8571351

[65] L. He , F. Michailidou , H. L. Gahlon , and W. Zeng , “Hair Dye Ingredients and Potential Health Risks From Exposure to Hair Dyeing,” Chemical Research in Toxicology 35, no. 6 (2022): 901–915, 10.1021/acs.chemrestox.1c00427. 35666914 PMC9214764

[66] B. Lah , T. Vidic , E. Glasencnik , T. Cepeljnik , G. Gorjanc , and R. Marinsek‐Logar , “Genotoxicity Evaluation of Water Soil Leachates by Ames Test, Comet Assay, and Preliminary *Tradescantia* Micronucleus Assay,” Environmental Monitoring and Assessment 139 (2008): 107–118, 10.1007/s10661-007-9819-7. 17566864

[67] M. Farré and D. Barceló , “Toxicity Testing of Wastewater and Sewage Sludge by Biosensors, Bioassays and Chemical Analysis,” TrAC, Trends in Analytical Chemistry 22, no. 5 (2003): 299–310, 10.1016/S0165-9936(03)00504-1.

[68] M. D. Hernando , A. R. Fernández‐Alba , R. Tauler , and D. Barceló , “Toxicity Assays Applied to Wastewater Treatment,” Talanta 65, no. 2 (2005): 358–366, 10.1016/j.talanta.2004.07.012. 18969807

[69] L. C. Gonçalves , M. M. Roberto , P. V. L. Peixoto , et al., “Toxicity of Beauty Salon Effluents Contaminated With Hair Dye on Aquatic Organisms,” Toxics 11, no. 11 (2023): 911, 10.3390/toxics11110911. 37999563 PMC10674561

[70] H. Shi , R. Magaye , V. Castranova , and J. Zhao , “Titanium Dioxide Nanoparticles: A Review of Current Toxicological Data,” Particle and Fibre Toxicology 10 (2013): 1–33, 10.1186/1743-8977-10-15. 23587290 PMC3637140

[71] Y. Li , J. Yan , W. Ding , Y. Chen , L. M. Pack , and T. Chen , “Genotoxicity and Gene Expression Analyses of Liver and Lung Tissues of Mice Treated With Titanium Dioxide Nanoparticles,” Mutagenesis 32, no. 1 (2017): 33–46, 10.1093/mutage/gew065. 28011748

[72] X. Jia , S. Wang , L. Zhou , and L. Sun , “The Potential Liver, Brain, and Embryotoxicity of Titanium Dioxide Nanoparticles on Mice,” Nanoscale Research Letters 12, no. 1 (2017): 478, 10.1186/s11671-017-2242-2. 28774157 PMC5540742

[73] A. C. Martins, Jr. , L. F. Azevedo , C. C. S. Rocha , et al., “Evaluation of Distribution, Redox Parameters, and Genotoxicity in Wistar Rats Co‐Exposed to Silver and Titanium Dioxide Nanoparticles,” Journal of Toxicology and Environmental Health, Part A 80 (2017): 1156–1165, 10.1080/15287394.2017.1357376. 28891756

[74] A. Papp , T. Horváth , N. Igaz , et al., “Presence of Titanium and Toxic Effects Observed in Rat Lungs, Kidneys, and Central Nervous System In Vivo and in Cultured Astrocytes In Vitro on Exposure by Titanium Dioxide Nanorods,” International Journal of Nanomedicine 15 (2020): 9939–9960, 10.2147/IJN.S275937. 33376320 PMC7765755

[75] Z. Shajari , A. Golestani , and A. Nowrouzi , “Acute Cytotoxic Effects of Titanium Dioxide (TiO_2_) and Chronic Exposure to Safe Dose Nanoparticle in the Hepatocarcinoma (HepG2) Cell Line,” Nanomedicine Research Journal 6, no. 3 (2021): 269–278, 10.22034/nmrj.2021.03.007.

[76] R.‐R. Zhu , S.‐L. Wang , R. Zhang , X.‐Y. Sun , and S.‐D. Yao , “A Novel Toxicological Evaluation of TiO_2_ Nanoparticles on DNA Structure,” Chinese Journal of Chemistry 25, no. 7 (2007): 958–961, 10.1002/cjoc.200790186.

[77] C. Ling , H. An , L. Li , et al., “Genotoxicity Evaluation of Titanium Dioxide Nanoparticles In Vitro: A Systematic Review of the Literature and Meta‐Analysis,” Biological Trace Element Research 199 (2021): 2057–2076, 10.1007/s12011-020-02311-8. 32770326

[78] M. P. Galiotte , P. Kohler , G. Mussi , and G. J. F. Gattás , “Assessment of Occupational Genotoxic Risk Among Brazilian Hairdressers,” Annals of Occupational Hygiene 52, no. 7 (2008): 645–651, 10.1093/annhyg/men037. 18596021

[79] G. M. Machado‐ Santelli and F. Siveiro , “Mutagênese e Carcinogênese,” in Fundamentos de Toxicologia, ed. Seizi Oga , Márcia Maria de Almeida Camargo , and José Antonio de Oliveira Batistuzzo (Atheneu, 2014), 71–84.

[80] M. M. Roberto and C. A. Christofoletti , “How to Assess Nanomaterial Toxicity? An Environmental and Human Health Approach,” in Nanomaterials—Toxicity, Human Health and Environment, ed. Simona Clichici , Gabriela Adriana Filip , and Gustavo Morari Do Nascimento (IntechOpen, 2019), 10.5772/intechopen.88970.

[81] M. Fenech , “Cytokinesis‐Block Micronucleus Cytome Assay,” Nature Protocols 2, no. 5 (2007): 1084–1104, 10.1038/nprot.2007.77. 17546000

[82] J. H. Houtgraaf , J. Versmissen , and W. J. van der Giessen , “A Concise Review of DNA Damage Checkpoints and Repair in Mammalian Cells,” Cardiovascular Revascularization Medicine 7, no. 3 (2006): 165–172, 10.1016/j.carrev.2006.02.002. 16945824

[83] C. Betoli , J. M. Skotheim , and R. A. M. de Bruin , “Control of Cell Cycle Transcription During G1 and S Phases,” Nature Reviews Molecular Cell Biology 14, no. 8 (2013): 518–528, 10.1038/nrm3629. 23877564 PMC4569015

[84] E. Lorge , M. Hayashi , S. Albertini , and D. Kirkland , “Comparison of Different Methods for an Accurate Assessment of Cytotoxicity in the In Vitro Micronucleus Test: I. Theoretical Aspects,” Mutation Research, Genetic Toxicology and Environmental Mutagenesis 655, no. 1–2 (2008): 1–3, 10.1016/j.mrgentox.2008.06.003. 18602494

[85] M. D. Fellows , M. R. O. O'Donovan , E. Lorge , and D. Kirkland , “Comparison of Different Methods for an Accurate Assessment of Cytotoxicity in the In Vitro Micronucleus Test II: Practical Aspects With Toxic Agents,” Mutation Research 655 (2008): 4–21, 10.1016/j.mrgentox.2008.06.004. 18602493

