# Supplementary material for: Untreated Hair Dye Effluents Enter the Environment: Are They a Threat to Human Health?
Source: Environ Toxicol. 2026 Feb 2;41(10):998–1010. doi: 10.1002/tox.70034 (PMC13537347; doi:10.1002/tox.70034)
Supplement: Supplementary file 1 [file TOX-41-998-s001.docx]

**Supp.Table 1** - Statistical data related to the cell viability assessment (Figure 1 - MTT and Trypan Blue tests) on HepG2/C3A cells exposed to beauty salon effluents.

| **Comparisons** | **MTT test**  **P Value^a^** | **Trypan Blue test**  **P Value^b^** |
| --- | --- | --- |
| NC vs. PC | <0.0001 | <0.0001 |
| NC vs. CE-1 | <0.0001 | <0.0001 |
| NC vs. CE-2 | 0.0001 | <0.0001 |
| NC vs. CE-3 | 0.0227 | <0.0001 |
| NC vs. CE-4 | 0.1182 | <0.0001 |
| NC vs. CE-5 | 0.2092 | 0.0003 |
| NC vs. CE-6 | >0.9999 | 0.0062 |
|  |  |  |
| NC vs. PC | <0.0001 | <0.0001 |
| NC vs. DE-1 | <0.0001 | <0.0001 |
| NC vs. DE-2 | <0.0001 | <0.0001 |
| NC vs. DE-3 | <0.0001 | <0.0001 |
| NC vs. DE-4 | 0.0618 | <0.0001 |
| NC vs. DE-5 | 0.1261 | <0.0001 |
| NC vs. DE-6 | 0.3385 | 0.0037 |

^a^Kruskal-Wallis/Dunn. ^b^ANOVA/Dunnett. NC: Negative control; PC: Positive control; CE-1: raw sample (100%); CE-2: sample diluted by 50.00%; CE-3: sample diluted by 25.00%; CE-4: sample diluted by 12.50%; CE-5: sample diluted by 6.25%; CE-6: sample diluted by 3.125%; DE-1: raw sample (100%); DE-2: diluted by 50.00%; DE-3: sample diluted by 25.00%; DE-4: sample diluted by 12.50%; DE-5: sample diluted by 6.25%; DE-6: sample diluted by 3.125%.
